# Predicting disordered regions in proteins using the profiles of amino acid indices

**DOI:** 10.1186/1471-2105-10-S1-S42

**Published:** 2009-01-30

**Authors:** Pengfei Han, Xiuzhen Zhang, Zhi-Ping Feng

**Affiliations:** 1 School of Computer Science and IT, RMIT University, Melbourne, VIC 3001, Australia; 2 The Walter and Eliza Hall Institute of Medical Research, Parkville, VIC 3050, Australia

## Abstract

**Background:**

Intrinsically unstructured or disordered proteins are common and functionally important. Prediction of disordered regions in proteins can provide useful information for understanding protein function and for high-throughput determination of protein structures.

**Results:**

In this paper, algorithms are presented to predict long and short disordered regions in proteins, namely the long disordered region prediction algorithm DRaai-L and the short disordered region prediction algorithm DRaai-S. These algorithms are developed based on the Random Forest machine learning model and the profiles of amino acid indices representing various physiochemical and biochemical properties of the 20 amino acids.

**Conclusion:**

Experiments on DisProt3.6 and CASP7 demonstrate that some sets of the amino acid indices have strong association with the ordered and disordered status of residues. Our algorithms based on the profiles of these amino acid indices as input features to predict disordered regions in proteins outperform that based on amino acid composition and reduced amino acid composition, and also outperform many existing algorithms. Our studies suggest that the profiles of amino acid indices combined with the Random Forest learning model is an important complementary method for pinpointing disordered regions in proteins.

## Background

Proteins are linear chains composed of 20 amino acids (aa), also called residues when they form chains by detaching water molecules, linked together by polypeptide bonds and folded into complex three-dimensional (3D) structures. Disordered regions (DRs) in protein sequence are structurally flexible and usually have low sequence complexity [1-4]. Physicochemically, DRs are enriched in charged or polar amino acids, and depleted in hydrophobic amino acids [5-7]. Proteins containing long DRs are called intrinsically unstructured or disordered proteins (IUPs or IDPs).

A number of protein disorder predictors have been developed by several groups, such as PONDR [8], RONN [9,10], DisProt [11,12], NORSp [13,14], DISpro [15], DISOPRED and DISOPRED2 [16,17], DisEMBL [18], IUPred [19], DRIP-PRED [20] and Spritz [21], and more recently DisPSSMP [22], VSL1 and VSL2 [23,24], POODLE-L [25], POODLE-S [26], Ucon [27], PrDOS [28] and metaPrDOS [29]. Most existing predictors are based on the Neural Network and Support Vector Machine learning models. The features used to construct the prediction models include amino acid composition (AAC) or reduced amino acid composition (RAAC) combined with the physiochemical properties of amino acids including aromaticity, net charge, flexibility, hydropathy, coordination number and sequence complexity [8-10]. To achieve high prediction accuracy, typically algorithms use many features as input. Some algorithms are based on the sequence alignment scores from PSI-BLAST or protein secondary structure information [16,17,21]. Either approach lowers the efficiency of these algorithms and hinders their application in high-throughput analysis.

It has been shown that short disordered regions have different characteristics from long disordered regions [30]. Algorithms perform well in predicting long disordered regions rarely perform well in predicting short disordered regions. In this paper, algorithms for predicting short and long DRs are developed separately based on the Random Forest learning model [31] and the profiles of the amino acid indices. The algorithm for long disordered regions, DRaai-L, can achieve an area of 85.1% under the receiver operating characteristic (ROC) curves in the 10 fold cross validation test. The algorithm targeting all kinds of disordered regions, DRaai-S, can achieve an area of 81.2% under the ROC curve in the 10 fold cross validation test and about 72.2% in the blind test on CASP7 targets. Both DRaai-L and DRaai-S achieve higher prediction accuracy as well as higher computation efficiency than many existing algorithms, which make them efficient tools for high-throughput prediction of disordered regions in proteins.

### Training and test data

In this study, the training data is derived from DisProt (version 3.6) [32] and PDB-Select-25 (the Oct.2004 version) [33]. DisProt is a collection of disordered regions of proteins based on published literature descriptions. It has 472 proteins entries and 1121 disordered regions. Only long disordered regions (>30aa) in DisProt3.6 are used to train DRaai-L, and it is denoted as *DL-train *hereafter. All disordered regions in DisProt3.6 were used to train DRaai-S, and it is denoted as *DS-train *hereafter. The ordered training data is extracted from PDB-Select-25, a representative set of protein data bank (PDB) chains that shows less than 25% sequence homology. We selected 366 high-resolution (< 2 Å) segments with well-defined structures which has no missing backbone or side chain coordinates and contains at least 80 residues. This collection of ordered training set includes a total of 80324 residues, and is referred to as *O-train *hereafter. The CASP7 targets were used as an independent test dataset to blind test the performance of prediction. The disorder contents of CASP7 are very different from those of DisProt3.6. The CASP7 dataset contains 96 sequences with a total of 19,891 residues, where only 170 disordered regions, or 1,189 (6%) residues are annotated as disordered. There is a significant amount (28% in aa) of short disordered regions containing 1 or 2 aa, and only 4 are long DRs of >30 aa (<2% in aa). While DisProt3.6 contains 352 regions of >30 aa with 47251 aa in total (36% in aa).

### The amino acid indices and feature selection

The amino acid index (AA-index) database AAindex [34] is a database of numerical indices representing various physiochemical and biochemical properties of amino acids or pairs of amino acids. Especially the AAindex1 database comprises 544 sets of numerical indices for the 20 amino acids, and all of them are derived from published literature.

The AA-indices that are highly correlated with the disordered or ordered status of the residues in the training protein sequences were used to construct the prediction model in our studies. The process of choosing these indices was implemented in three steps. First of all, given a set of indices and a training sequence, the training sequence is transformed into two vectors $\vec{V}1$ and $\vec{V}2$. $\vec{V}1$ is generated by replacing ordered and disordered resides with the number -1 and 1 respectively based on the annotations from the databases. $\vec{V}2$ is the result by substituting the amino acid code by the corresponding AA-index value.

Note that as different sets of AA-index are of different scales in the AA-index database, the Z-transformation (${P^{\prime }}_{r}$) is applied for each set of index before the substitution. For a set of AA-index, the *Z*-transformation is shown in Equation 1.

(1) $${P^{\prime }}_{r}=\frac{P_{r}-\bar{P}}{\sigma }$$

*P*_*r *_represents an AA-index value and *r *varies for the 20 amino acids denoted as 1..20. $\bar{P}$ and *σ *are the mean and standard deviation of the 20 AA-index values:

(2) $$\bar{P}=\frac{\sum_{r=1}^{20}P_{r}}{20}$$

and

(3) $$\sigma =\sqrt{\frac{1}{20}\sum_{r=1}^{20}{\left(P_{r}-\bar{P}\right)}^{2}}$$

After the AA-index substitution, the structural influence to a residue by its surroundings is calculated using a smooth function. The Savitzky-Golay filter [35] is used to smooth both $\vec{V}1$ and $\vec{V}2$ in our study with a window of 17 aa. This filter essentially performs a polynomial regression on the $\vec{V}1$ and $\vec{V}2$ to determine the smoothed value for each point. The main advantage of Savitzky-Golay is to preserve features of the distribution such as relative max score, min score and width of disordered or ordered regions, which are usually "flattened" by other smooth techniques. The smoothed vectors $\vec{V}1^{\prime }$ and $\vec{V}2^{\prime }$ denote the results of filtering $\vec{V}1$ and $\vec{V}2$ respectively.

Finally the correlation coefficient of an AA-index set and a protein sequence is calculated as shown in Equation 4, where *N *represents the length of the sequence under consideration.

(4) $$R_{\vec{V}1^{\prime }\vec{V}2^{\prime }}=\frac{\sum_{r=1}^{N}\left(\vec{V}1^{\prime }-\bar{\vec{V}}1^{\prime }\right)\left(\vec{V}2^{\prime }-\bar{\vec{V}}2^{\prime }\right)}{\left(N-1\right)\sigma_{\vec{V}1},\sigma_{\vec{V}2^{\prime }}}$$

The correlation coefficient $R_{\vec{V}1^{\prime }\vec{V}2^{\prime }}$ is in the range [-1..1]. A positive coefficient indicates that the set of AA-indices is positively correlated with the order/disorder status of residues in the sequence, whereas a negative coefficient indicates negative correlation.

The sets of AA-indices that are mostly related to the disorder/order status of residues in all our training sequences were used to construct the prediction model. Specifically these sets of indices were chosen so that

• To maximize the summarization of the absolute correlation coefficients of the index over all training sequences.

• To maximize the number of protein sequences that the index uniformly correlates with.

Based on the above two criteria, the top 40 AA-index sets were selected. Among the 40 sets, many are highly correlated (with correlation coefficient of at least 0.8), and as a result five representative index sets were selected, as shown in Table 1.

**Table 1:** Amino acid indices related to (dis)order. The five sets of amino acid indices that are most correlated to the (dis)order of proteins are the features used in prediction.

AA-index set	Description
VINM940102	Normalized flexibility parameters (B-values) for each residue surrounded by none rigid neighbours
BULH740101	Surface tension of amino acid solutions: A hydrophobicity scale of the amino acid residues
PUNT030102	Knowledge-based membrane-propensity scale from 3D_Helix in MP- topo databases
CHOP780203	Normalized frequency of beta-turn
CHOP780211	Normalized frequency of C-terminal non beta region

From the description of these 5 sets of AA-indices listed in Table 1, we can see that they are strongly correlated with protein structures. For example, index BULH740101 represents hydrophobicity while it is known that ordered regions tend to be hydrophobic, indices CHOP780203 and CHOP780211 represent alpha and turn propensities which has been widely used in secondary structure prediction.

### The Moreau-Broto autocorrelation functions of AA-indices

The profiles of amino acid indices along a protein sequence have been used in the protein structural and functional classification studies [36-38]. Given an AA-index set, the normalized Moreau-Broto autocorrelation coefficient for an amino acid protein sequence is defined in Equation 5:

(5) $$AC(d)=\frac{1}{N-d}\times \sum_{i=1}^{N-d}P_{i}P_{i+d}$$

where *N *is the length of the sequence under consideration, and *d *is an integer larger than zero and describe the lag of the autocorrelation or the distance in the number of residues separated in the protein sequence. In this study, *d *is set to 1..30. *P*_*i *_and *P*_*i*+*d *_are the AA-index values at positions *i *and *i *+ *d *normalized by Z-transformation respectively. We used the Moreau-Broto autocorrelation functions generated from smoothed vector $\vec{V}2^{\prime }$ under different windows as input to develop the DRaai-L algorithm, and used the vector $R_{\vec{V}1^{\prime }\vec{V}2^{\prime }}=\frac{\sum_{r=1}^{N}\left(\vec{V}1^{\prime }-\bar{\vec{V}}1^{\prime }\right)\left(\vec{V}2^{\prime }-\bar{\vec{V}}2^{\prime }\right)}{\left(N-1\right)\sigma_{\vec{V}1},\sigma_{\vec{V}2^{\prime }}}$ directly to develop the DRaai-S algorithm.

## Methods

The Random Forest machine learning model is the underlying model in this study. A random forest is an ensemble of unpruned decision trees, where each tree is grown using a (bootstrap) subset of the training dataset [39]. Bootstrap is the training set drawn randomly from the original training set with an equal number of training samples. Each tree induced from bootstrap samples grows to full length and the number of trees in the forest is adjustable. After training, every path from the root of a tree to a leaf gives one if-then rule and can be used for prediction. As an ensemble machine learning model the random forest has no risk of overfitting with an increasing number of trees. However, after certain point, the increase of number of trees leads to trivial improvement of prediction accuracy while prolonging the time of training and prediction significantly. The random forest implementation of the WEKA data mining package [40] is used to build our models.

### DRaai-L: predicting long DRs using AA-indices

*DL-train *and *O-train *are used to train the algorithm DRaai-L. For each ordered or disordered region in the *DL-train *and *O-train *datasets, a window of *w *aa (by default *w *= 31) slides along a sequence from N-terminus to C-terminus one residue at a time. The Moreau-Broto autocorrelation of the 5 sets of AA-indices in each window is calculated with *d *assigned from 1..30. So *n *= 5 × 30 = 150 elements are generated from a window. When a window of *w *residues slides along a protein sequence of *L*_*i *_residues, the sequence is represented by (*L*_*i*_/*w*) × *n *elements. These elements are used as the input parameters to the random forest to train the DRaai-L model.

For a query sequence, a window slides along the sequence and its corresponding vectors $\vec{V}2$' is computed using the Moreau-Broto autocorrelation functions. The smoothed vectors $\vec{V}2$' are then input to the DRaai-L model, and the disordered/ordered status of each residue is predicted.

### DRaai-S: predicting short DRs using AA-indices

All disordered regions in DisProt3.6, *DS-train*, were used to train DRaai-S. Each amino acid sequence in the training set was replaced with numerical sequences by the 5 sets of AA-indices and smoothed using the Savitzky-Golay filter (with a window of 17 aa). Then the smoothed vectors $\vec{V}2$' are directly used as input parameters to develop the DRaai-S model,

To predict the disorder of a query sequence, the sequence is transformed similarly to the 5 smoothed vectors $\vec{V}2$', and then they are input to the DRaai-S model to predict the disorder/order of each residue.

## Evaluation

The distribution of ordered/disordered residues are very imbalanced in both DisProt3.6 and CASP7. With the fact that disordered residues are by far the minority in both databases, overall accuracy (Q2) is not a good measure to evaluate disorder prediction algorithms [41]. Ideally a disorder algorithm should be highly sensitive on disordered regions while not producing many false positive predictions. The confusion matrix of an algorithm, which comprises True Positive (TP), False Positive (FP), True Negative (TN) and False Negative (FN), can be used to evaluate the performance of the algorithm. Note that in the context of disorder prediction P and N are the total number of labelled disordered and ordered residues respectively.

The receiver operating characteristic (ROC) curves were used to evaluate the prediction accuracy. Each point of a ROC curve is defined by a pair of values for the false positive rate (x = FP/N) and the true positive rate (y = TP/P). For a prediction algorithm, by adjusting the parameters, the true positive rate can be plotted under different false positive rates and a smooth ROC curve can be obtained.

The performance of DRaai-L and DRaai-S is measured in different methods as described below.

• The Sensitivity is the true positive rate, which is the percentage of residues correctly predicted as disordered in relation to the total number of actual disordered residues.

• The Precision is the percentage of true positives in relation to the total number of predicted positives.

• The Specificity is the percentage of residues correctly predicted as ordered in relation to the total number of ordered residues. The false positive rate is 1-Specificity.

• *S*_*product *_is a single measurement combining sensitivity and specificity: *S*_*product *_= Sensitivity × specificity. *S*_*product *_favours disorder prediction.

• The Matthew Correlation Coefficient (MCC) ranges between -1 and +1, and favors correct predictions of disordered residues. MCC is defined as

$\frac{TP\times TN-FP\times FN}{\sqrt{\left(TP+FP\right)\times \left(TP+FN\right)\times \left(TN+FP\right)\times \left(TN+FN\right)}}.$

• *S*_*w *_is a measurement that assigns class weights that are reversely related to class distribution. As a result, *S*_*w *_rewards models for correctly predicting a disordered residue. *S*_*w *_was used in assessing the prediction of disordered residues in CASP6 and CASP7. *S*_*w *_is defined as

$\frac{W_{disorder}\times TP-W_{order}\times FP+W_{order}\times TN-W_{disorder}\times FN}{W_{disorder}\times P+W_{order}\times N},$

where *W*_*disorder *_and *W*_*order *_are the weights for disorder and order respectively. *W*_*disorder *_and *W*_*order *_should be set to be inversely proportional to the disorder and order content in the data under consideration. For evaluation on DisProt3.6, *W*_*disorder *_= 85 and *W*_*order *_= 15. For evaluation on on CASP7, *W*_*disorder *_= 94 and *W*_*order *_= 6.

The random forest package we use provides the out-of-bag test to estimate prediction error rate using data randomly withheld from each iteration of tree development. However this approach significantly overestimates the performance when a window technique is used.

The performance of both DRaai-L and DRaai-S are evaluated on DisProt3.6 using 10-fold cross validation. The performance of DRaai-S is further evaluated by blind test on CASP7 targets.

DRaai-L and DRaai-S are compared with algorithms based on the Random Forest model but constructed using the amino acid composition (AAC) and reduced AAC (RAAC) [42] information of the primary sequences. They are also compared with other existing disorder prediction algorithms.

## Results 

The results of evaluating DRaai-L and DRaai-S using 10-fold cross validation tests on DisProt3.6 and blind test on CASP7 are presented separately.

### The performance of DRaai-L

**Figure 1:**
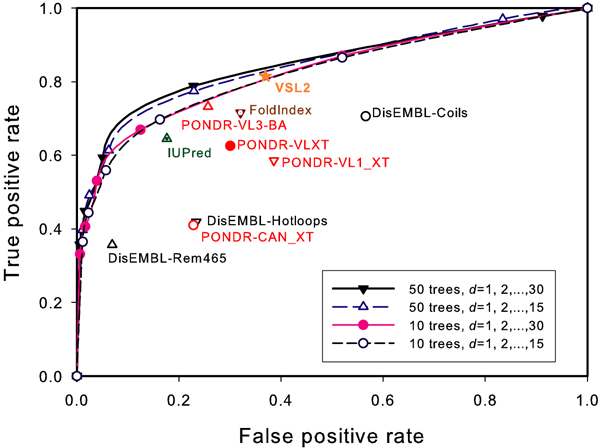
**Performance of DRaai-L**. The ROC curves of DRaai-L in 10-fold cross validation test. All independent points in the figure are results obtained from the respective online predictors with their default settings.

The performances of DRaai-L under different number of trees for the random forest model and different *d *values for the Moreau-Broto autocorrelation coefficients are presented using ROC curves shown in Figure 1. The area under the ROC for the model trained with 50 trees and the auto-correlation coefficients generated from *d *= 1, 2,, 30 aa is 85.1%. Even for the model trained with 10 trees and the auto-correlation coefficients generated from *d *= 1, 2,...,15 aa, the area under the ROC can reach 82.7%. This result is better than that trained with AAC (78.6%, under 50 trees and *d *= 1, 2,...,30) or RAAC (74.1%, under 10 trees and *d *= 1, 2,...,15). This result is also better than that of most other available algorithms, as indicated by the separate points in Figure 1.

Table 2 describes the performance of DRaai-L in comparison with other published algorithms. The performance is measured in terms of Sensitivity, Precision, Specificity, *S*_*product*_, MCC and *S*_*w*_. DRaai-L is with a setting of 50 trees and *d *= 1, 2,...,30. With these six methods of evaluation, the performance of DRaai-L is just below IUPred, but better than most other predictors.

**Table 2:** The performance DRaai-L on DisProt3.6. The performance of DRaai-L in the independent test on 10% of DisProt3.6 targets under various measures in comparison with other predictors.

Algorithm	Sensitivity	Precision	Specificity	*S*_*product*_	MCC	*S*_*w*_
DisEMBL(Coil)	0.71	0.33	0.43	0.31	0.13	0.24
DisEMBL(Rem465)	0.36	0.67	0.93	0.33	0.36	0.29
DisEMBL(Hot Loop)	0.42	0.41	0.77	0.32	0.18	0.19
FoldIndex	0.72	0.46	0.68	0.49	0.36	0.40
IUPred	0.65	0.59	0.82	0.53	0.46	0.47
PONDR(CANXT)	0.41	0.41	0.77	0.32	0.18	0.18
PONDR(VL)	0.55	0.55	0.77	0.42	0.32	0.29
PONDR(VLXT)	0.63	0.45	0.70	0.44	0.30	0.33
PONDR(XL)	0.59	0.37	0.61	0.36	0.18	0.20
VSL2	0.76	0.79	0.79	0.60	0.55	0.55
**DRaai-L**	**0.78**	**0.80**	**0.80**	**0.62**	**0.58**	**0.57**

### The performance of DRaai-S

**Figure 2:**
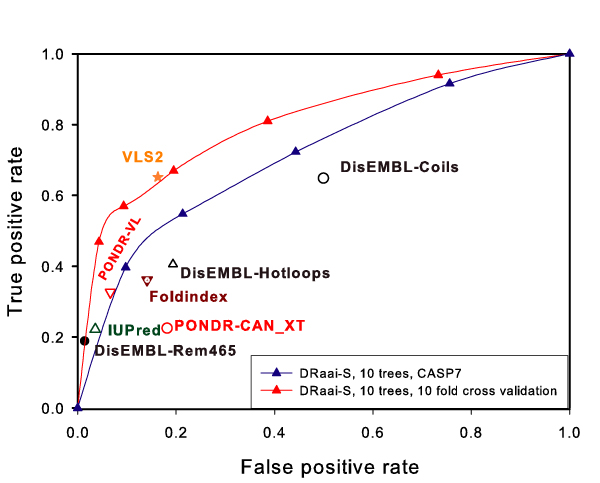
**Performance of DRaai-S**. The ROC curves of DRaai-S in 10-fold cross validation test and blind test on CASP7. All independent points in the figure are results on CASP7 targets obtained from the respective online predictors with their default settings.

Figure 2 shows the ROC curves for DRaai-S under 10 fold cross validation and on CASP7 targets. The area under ROC of DRaai-S in 10 fold cross validation is 81.2%, while it dropped to 72.2% when used to predict the CASP7 targets. Table 3 describes the performance of DRaai-S on CASP7 in comparison with other predictors. DRaai-S is with a setting of 10 trees and a smoothing window of 17 aa. The results in both Figure 2 and Table 3 demonstrate that DRaai-S can achieve comparable or even more accurate prediction than some published algorithms.

**Table 3:** The performance DRaai-S on CASP7. The performance of DRaai-S of independent test on CASP7 targets under various measures in comparison with other predictors.

Algorithm	Sensitivity	Precision	Specificity	Sproduct	MCC	Sw
DisEMBL(Coil)	0.65	0.08	0.50	0.33	0.07	0.15
DisEMBL(Rem465)	0.19	0.47	0.99	0.19	0.27	0.18
DisEMBL(Hot Loop)	0.41	0.12	0.81	0.33	0.12	0.21
FoldIndex	0.36	0.14	0.86	0.31	0.14	0.22
IUPred	0.22	0.28	0.96	0.21	0.21	0.19
PONDR(CANXT)	0.23	0.07	0.82	0.18	0.03	0.05
PONDR(VL)	0.33	0.24	0.93	0.30	0.23	0.26
PONDR(VLXT)	0.46	0.12	0.79	0.36	0.14	0.25
PONDR(XL)	0.30	0.06	0.72	0.22	0.01	0.02
VSL2	0.73	0.21	0.85	0.61	0.33	0.58
**DRaai-S**	**0.55**	**0.14**	**0.79**	**0.43**	**0.19**	**0.34**

In summary, by using the simple AA-index information, both DRaai-L and DRaai-S have shown better performance than many well developed published algorithms. DRaai-L and DRaai-S have the potential to be further improved by adjusting the sets of AA-indices, the number of residues to be smoothed, and the number of residues considered in the auto-correlation function.

## Discussion

The good performance of DRaai-L compared with the other published algorithms shown in Figure 1 and Table 2 indicates that the continuous correlations among the nearby residues along a primary sequence implies ordered/disordered structural information. It is well known that the residues involved in ordered structures are always close to other residues in space. In other words, they are constrained by backbone or side chain interactions from other residues, and hence they have higher density in the contact map [27]. Indeed the auto-correlation functions used in DRaai-L reflect such contact information. If the residues in a fragment of more than 30 aa do not show any kind of correlation between each other, it is very unlikely that these residues are constrained by each other or form stable contacts, they therefore have high propensity to be disordered.

The prediction results of DRaai-S on DisProt3.6 and CASP7 shown in Figure 2 and Table 3 indicate that the position specific profiles of the physiochemical properties of residues determine whether they are involved in short disordered regions. The poor performance of DRaai-S compared with DRaai-L indicates that accurately predicting short disordered regions is significantly more challenging than predicting long disordered regions. This is partially due to the difficulty of extracting local sequence information, but more importantly due to the lack of sufficient robust short disordered regions in the training dataset. Therefore, a short DR predictor trained from very limited number of short disordered regions can produce a high false positive rate or fluctuated prediction accuracy.

CASP targets are a typical set of highly ordered globular proteins that are suitable for protein structural determination by either NMR or X-crystallography. As such the distribution of disorder in CASP targets is not a typical representation of disorder in all proteomes. Indeed the distribution of short DRs in DisProt3.6 is significantly different. Among the limited number of disordered regions in CASP targets, the majority are either very short or distributed in the terminal regions. However protein sequence-structural relationship in the terminal regions has not been well established [43]. As a result the disordered regions in CASP targets are extremely difficult to predict. To improve the prediction accuracy on CASP targets, many existing prediction algorithms use various features including predicted secondary structure and position specific scoring matrix, which typically requires lengthy PSI-BLAST search. DRaai-S uses the simple and uniform AA-index information and can efficiently predict disordered regions in CASP targets, with a reasonable accuracy that has a great promise to be further improved.

## Conclusion

Protein disorder studies are becoming increasingly important because IUPs are common and functionally important. Experimental studies of IUPs are expensive and time consuming. In this paper we have presented two algorithms DRaai-L and DRaai-S for predicting disordered regions in proteins, using the profiles of AA-indices and the Random Forest machine learning model. By using Moreau-Broto auto-correlation functions and profiles of AA-indices and Savitzky-Golay filter, long disordered regions and short disordered regions can be accurately predicted with DRaai-L and DRaai-S respectively.

With the simple and uniform AA-index information, both DRaai-L and DRaai-S outperform some well developed algorithms, with high computing efficiency. This makes them competitive tools to be used in large-scale structural analyses and in comparative proteome studies.

## List of abbreviations used

aa: amino acid; AAC: amino acid composition; AA-index: amino acid index; DR: disordered region; IDP: intrinsically disordered protein; IUP: intrinsically unstructured protein; RAAC: reduced amino acid composition; ROC: receiver operating characteristic.

## Competing interests

PH is supported by an Australian Postgraduate Award. XZ is supported in part by an RMIT Emerging Researcher Grant. ZPF is supported by an APD Award from the Australian Research Council.

## Authors' contributions

PH carried out the algorithm implementation and performance evaluation. XZ and ZPF participated in the design of the study, and drafted the manuscript.
